# Evaluation of the Safety and Efficacy of Avacopan, a C5a Receptor Inhibitor, in Patients With Antineutrophil Cytoplasmic Antibody–Associated Vasculitis Treated Concomitantly With Rituximab or Cyclophosphamide/Azathioprine: Protocol for a Randomized, Double-Blind, Active-Controlled, Phase 3 Trial

**DOI:** 10.2196/16664

**Published:** 2020-04-07

**Authors:** Peter A Merkel, David R Jayne, Chao Wang, Jan Hillson, Pirow Bekker

**Affiliations:** 1 Division of Rheumatology Department of Medicine University of Pennsylvania Philadelphia, PA United States; 2 Division of Clinical Epidemiology Department of Biostatistics, Epidemiology, and Informatics University of Pennsylvania Philadelphia, PA United States; 3 Department of Medicine University of Cambridge Cambridge United Kingdom; 4 Biostatistics Pharma Data Associates, LLC Piscataway, NJ United States; 5 Research and Development ChemoCentryx, Inc Mountain View, CA United States

**Keywords:** ANCA-associated vasculitis, avacopan, C5a receptor, ADVOCATE

## Abstract

**Background:**

Antineutrophil cytoplasmic antibody (ANCA)–associated vasculitis is a serious, often life-threatening disease. In new-onset disease or a relapse, the standard treatment is immunosuppressive therapy with glucocorticoids; these therapies are associated with substantial short- and long-term toxicity. Complement component 5a (C5a) binding to C5a receptor (C5aR) may play a central role in the pathogenesis of ANCA-associated vasculitis. Avacopan is a novel, orally bioavailable, and highly selective antagonist of human C5aR. Avacopan does not interfere with the production of C5b or the membrane attack complex (ie, terminal complement complex) and does not block C5a binding to a second receptor, C5L2 (also called C5aR2), shown to be protective in antimyeloperoxidase glomerulonephritis. This trial will evaluate if avacopan replaces the need for chronic glucocorticoids in the treatment of ANCA-associated vasculitis.

**Objective:**

The aim of this study is to determine the proportions of patients in remission at week 26 and with sustained remission at week 52, defined as Birmingham Vasculitis Activity Score=0, and not taking glucocorticoids within the 4 weeks before week 26 and week 52, respectively.

**Methods:**

The Avacopan Development in Vasculitis to Obtain Corticosteroid elimination and Therapeutic Efficacy study is a randomized, double-blind, active-comparator (prednisone), 2-arm study evaluating the safety and efficacy of avacopan versus prednisone, administered in combination with other immunosuppressive therapy. Eligible subjects will have active disease requiring induction of remission. Subjects are stratified based on the type of immunosuppressive therapy, ANCA subtype, and new or relapsing disease. Target sample size is 300 patients, enrolled at over 200 sites globally. All authors and local ethics committees approved the study design. All patients will provide informed consent.

**Results:**

Enrollment of patients was completed in Q4 2018. Topline results are anticipated to be published by Q3 2020.

**Conclusions:**

Results will be released irrespective of whether the findings are positive or negative.

**Trial Registration:**

ClinicalTrials.gov NCT02994927; https://clinicaltrials.gov/ct2/show/NCT02994927

**International Registered Report Identifier (IRRID):**

DERR1-10.2196/16664

## Introduction

### Background

Antineutrophil cytoplasmic antibody (ANCA)–associated vasculitis is a serious, often life-threatening disease that includes three related forms of small-vessel vasculitis: granulomatosis with polyangiitis (GPA), microscopic polyangiitis (MPA), and eosinophilic granulomatosis with polyangiitis (Churg-Strauss). GPA and MPA are thought to be triggered by the production of circulating autoantibodies against the neutrophil-expressed antigens myeloperoxidase (MPO) or proteinase 3 (PR3). The disease may present with a wide spectrum of clinical manifestations of varying degrees of severity including but not limited to skin lesions, sinonasal, pulmonary inflammation or hemorrhage, and glomerulonephritis.

The proinflammatory complement C5a ligand and its receptor C5aR (also referred to as CD88) appear to play a central role in the pathogenesis of ANCA-associated vasculitis [1-4]. When stimulated by inflammatory cytokines, activation of the terminal C5a/C5aR axis generates an autoamplification loop that drives the acute necrotizing vasculitic process from primed neutrophils resulting from their interaction with ANCAs. C5a acting on C5aR is a potent neutrophil chemoattractant and agonist [5], which increases neutrophil adhesion, induces neutrophil degranulation, and produces reactive oxygen intermediates. Activation of C5aR also decreases neutrophils’ deformability, slowing their ability to traverse small blood vessels particularly in the presence of ANCA [6]. Moreover, C5a activates vascular endothelial cells through C5aR, promoting their retraction and increased vascular permeability [7,8].

The current standard treatments for induction of remission for GPA and MPA rely on substantial immunosuppression and consist primarily of glucocorticoids combined with either cyclophosphamide (intravenous or oral) followed by oral azathioprine, or rituximab [9-11]*.* Despite these available therapies, patients with ANCA-associated vasculitis have a 9-fold increased risk of mortality in the first year compared with healthy controls, attributed to infection, active vasculitis, and the effects of renal insufficiency [12,13]. More than 50% of the 1-year mortality is attributed to treatment-related adverse events rather than active vasculitis [12,13]. Furthermore, substantial accumulated permanent damage can occur when the disease is insufficiently controlled; for example, 15% to 38% of patients develop end-stage renal disease within 5 years [14-19].

Although glucocorticoids have a relatively rapid onset of action, their use is associated with an overall negative impact on the patient’s health and health-related quality of life (HRQoL), attributable to known acute and chronic effects of these medications [20]. In the treatment of ANCA-associated vasculitis, these include increased risks of infection [21-23], diabetes mellitus, fractures, gastrointestinal bleeding, hypertension, cataracts, and progressive organ damage [24]. Weight gain, sleep disturbance, lipodystrophy, and neuropsychiatric disturbances (including irritability, anxiety, depression, and hyperactivity) have also been reported [25,26]. Moreover, an analysis of data from patients enrolled in several trials conducted by the European Vasculitis Study Group showed that the level of damage associated with the use of glucocorticoids in ANCA-associated vasculitis increased with length of exposure [24]. In relapsing disease, the repeated use of glucocorticoids leads to even further damage. Collectively, the data indicate a need for effective glucocorticoid-sparing treatments that can help avoid complications associated with exposure to glucocorticoids and limit organ damage through more rapid and sustained disease control.

Avacopan (previously known as CCX168) is a novel, orally bioavailable, highly selective human C5aR antagonist that lacks any other known pharmacology [27]. Importantly, avacopan does not inhibit the interaction of C5a with the related receptor C5L2 (also referred to as C5aR2), thought to have anti-inflammatory properties [27] and also shown to impart protective effects in a mouse model of anti-MPO-induced glomerulonephritis [28]. As a specific C5aR antagonist, avacopan does not interfere with the formation of the terminal complement complex or membrane attack complex C5b-9, which is necessary for clearance of pathogenic encapsulated bacteria such as *Neisseria meningitidis*.

### Prior Work

Along with a favorable safety profile in a phase 1 dose escalation clinical trial in healthy volunteers, 30 mg of avacopan administered twice daily was shown to produce plasma concentrations that provided near complete inactivation of C5aR on blood neutrophils throughout the day, including ≥90% C5aR blockade at trough levels of the drug [27]. This exposure was sufficient to achieve optimal blockade of C5a-induced CD11b upregulation, as well as C5a-induced degranulation, reactive oxygen intermediate production, and C5a-directed migration of neutrophils ex vivo, forming the basis for the dose selected for avacopan in subsequent clinical studies of ANCA-associated vasculitis.

In a phase 2 clinical trial (NCT01363388) conducted in ANCA-associated vasculitis, 67 patients received one of the three treatments: avacopan with no prednisone, avacopan with low-dose (20 mg/day) prednisone (with taper), or placebo control with standard dose (60 mg/day) prednisone (with taper); treatment was administered along with either cyclophosphamide followed by azathioprine or rituximab. Avacopan was shown to replace glucocorticoids without compromising efficacy. Clinical response at week 12, defined as a 50% reduction in Birmingham Vasculitis Activity Score (BVAS) from baseline, was observed in 70% of the standard treatment group (placebo), 86% of the avacopan plus low-dose prednisone group (*P*=.002 for noninferiority, compared with standard care), 81% of the avacopan without prednisone group (*P*=.01 for noninferiority, compared with standard care), with a trend for faster control of disease in the avacopan groups relative to control [29]. Patients in the avacopan groups also had a rapid improvement in albuminuria (an indicator of preserved renal function), whereas the estimated glomerular filtration rate and hematuria improved in all treatment groups. Fewer glucocorticoid-associated adverse effects (primarily driven by a lower incidence of new-onset or worsening diabetes mellitus and psychiatric disorders) along with improvements in HRQoL (Medical Outcomes Study Short-Form 36 [SF-36] version 2, and EuroQOL [EQ-5D-5L]) were also associated with avacopan.

A second phase 2 trial of 12-week duration in 42 patients with ANCA-associated vasculitis (NCT02222155) was designed to examine the safety of avacopan when combined with standard of care therapy including glucocorticoids. Comparison of the avacopan versus placebo groups revealed that avacopan therapy when added to standard of care added no new safety signals beyond those associated with standard of care [30].

### Goals and Objectives of This Study

These phase 2 studies suggest that avacopan is well tolerated and that specific inhibition of the C5a/C5aR interaction on inflammatory cells may improve outcomes in ANCA-associated vasculitis, while reducing glucocorticoid exposure and their associated side effects observed with the standard of care. On the basis of these results, the underlying hypothesis for the phase 3 clinical trial protocol presented here (NCT02994927) is that avacopan is at least as effective as glucocorticoids in rapidly inducing and sustaining remission of signs and symptoms of ANCA-associated vasculitis when combined with either cyclophosphamide followed by azathioprine or with rituximab. As a selective anti-inflammatory agent, avacopan may also have safety advantages compared with glucocorticoids.

The primary objective of the trial is to evaluate the efficacy of avacopan to induce and sustain remission in patients with ANCA-associated vasculitis, when used in combination with immunosuppressive therapy, rituximab, or cyclophosphamide followed by azathioprine (or mycophenolate mofetil), but in the absence of months of treatment with glucocorticoids. As many patients in the trial are expected to have renal involvement due their vasculitis and methotrexate would be contraindicated in such patients, methotrexate was not an option for use following cyclophosphamide; this stipulation also aimed to reduce variation in the treatment groups. Disease remission is defined as achieving a BVAS score [31,32] of 0 (no evidence of active vasculitis) and no use of glucocorticoids for the treatment of ANCA-associated vasculitis within 4 weeks before week 26. Sustained remission is defined as remission at week 26 without relapse to week 52 (BVAS=0) and no use of glucocorticoids for ANCA-associated vasculitis within 4 weeks before week 52.

Secondary objectives include evaluation of the effect of treatment with avacopan versus standard of care on overall safety, glucocorticoid-related toxicity, rapidity of response, changes in HRQoL, changes in renal disease, and cumulative organ damage (vasculitis damage index).

## Methods

### Study Design

Enrollment of 300 patients is planned across more than 200 sites in 20 countries on 4 continents. Patients who drop out following randomization are not replaced. Briefly, after an explanation of the study and obtaining written informed consent, potentially eligible patients are screened against the inclusion and exclusion criteria (Textboxes 1 and 2) to confirm eligibility including review of their medical history.

**Major inclusion criteria for the randomization study.**

- Age ≥12 years
- Newly diagnosed or relapsing antineutrophil cytoplasmic antibody (ANCA)–associated vasculitis with granulomatosis with polyangiitis or microscopic polyangiitis
- Positive for antiproteinase 3 or antimyeloperoxidase ANCA
- Active disease, as assessed by ≥1 major item and ≥3 nonmajor items, or ≥2 renal items on Birmingham Vasculitis Activity Score (Multimedia Appendix 1) [31,32]
- Estimated glomerular filtration rate ≥15 mL/min/1.73 m^2^

Patients are required to fast for at least 9 hours before the first dose of study drug to allow for baseline low-density lipoprotein–cholesterol measurements. Upon arriving at the study site, patients undergo baseline assessments including sample collections to test for autoimmune serologies, hematologic parameters, a comprehensive chemistry panel, urinalysis, and additional blood and urine sampling for research purposes; HRQoL questionnaires (SF-36 and EQ-5D-5L) and glucocorticoid toxicity index [33] are completed.

**Major exclusion criteria for the randomization study.**

- Any of the following conditions: alveolar hemorrhage requiring invasive pulmonary ventilation support, other multisystem autoimmune disease (including eosinophilic granulomatosis with polyangiitis, lupus, IgA vasculitis (Henoch-Schönlein), rheumatoid vasculitis, Sjögren's syndrome, antiglomerular basement membrane disease, or cryoglobulinemic vasculitis)
- Requires dialysis or plasma exchange within 12 weeks before screening
- History of kidney transplant
- Immunosuppressive therapies: received cyclophosphamide ≤12 weeks before screening
- On azathioprine, methotrexate, or mycophenolate mofetil at screening and unwilling to discontinue use and switch to cyclophosphamide or rituximab on day 1
- Received intravenous glucocorticoids, >3000 mg methylprednisolone equivalent, within 4 weeks before screening or oral glucocorticoid >10 mg prednisone-equivalent within 6 weeks continuously before screening
- Received rituximab or other B-cell antibody ≤52 weeks before screening, or ≤26 weeks before screening provided B cell reconstitution has occurred, that is, CD19 count >0.01 × 109/L)
- Received antitumor necrosis factor treatment <12 weeks before screening
- For patients scheduled to receive cyclophosphamide: urinary outflow obstruction, active infection, or platelet count <50,000/μL before start of dosing
- Previous receipt of avacopan
- History of cancer within last 5 years, with the exception of excised basal cell or squamous cell carcinoma of the skin, or carcinoma in situ such as cervical or breast carcinoma in situ that has been excised or resected completely and is without evidence of local recurrence or metastasis
- Evidence of hepatic disease (transaminases, alkaline phosphatase >3 times upper limit of normal)
- Known active infection with tuberculosis, hepatitis B or C virus, or human immunodeficiency virus
- White blood cell count <3500/µL or neutrophil count <1500/µL, or lymphocyte count <500/µL at baseline

#### Stratification and Treatment Groups

**Figure 1:**
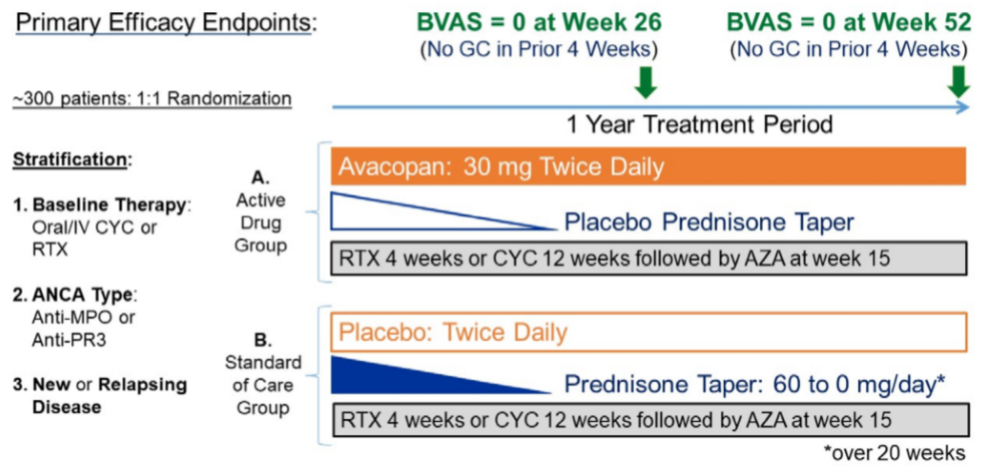
Avacopan Development in Vasculitis to Obtain Corticosteroid Elimination and Therapeutic Efficacy trial design. AZA: azathioprine; BVAS: Birmingham Vasculitis Activity Score; CYC: cyclophosphamide; GC: glucocorticoids; IV: intravenous; MPO: myeloperoxidase; PR3: proteinase 3; RTX: rituximab.

Eligible patients with ANCA-associated vasculitis are stratified according to 3 parameters: (1) baseline immunosuppressive therapy (oral cyclophosphamide, intravenous cyclophosphamide, or rituximab), (2) type of ANCA (anti-PR3 or anti-MPO), and (3) whether the patient is newly diagnosed or has relapsing disease (Figure 1). Following stratification, patients are randomized in a 1:1 ratio to 2 treatment groups receiving baseline immunosuppressive therapy plus either (A) blinded avacopan 30 mg twice daily plus placebo that matches prednisone or (B) blinded placebo that matches avacopan plus prednisone starting at 60 mg/day (or adjusted for weight). In the (B) group (standard of care), study-supplied prednisone is tapered to zero by day 140 (Table 1). Additional (nonstudy supplied) glucocorticoids are allowed before enrollment and during the first 4 weeks if required for initial control of disease as outlined later (*Additional Glucocorticoids*). In addition, the protocol allows for the use of low-dose glucocorticoids (up to 10 mg/day prednisone or equivalent) to treat adrenal insufficiency or to treat worsening or relapsing disease. A minimization algorithm is used to dynamically assign patients to a treatment group, which seeks to balance treatment groups with respect to each stratification factor. In adolescents (aged 12-17 years), the initial dose of avacopan can be adjusted downward (to 10 or 20 mg twice daily) depending on body weight with potential refinement based on plasma levels of avacopan. Any dose changes are determined by an unblinded reviewer, not otherwise associated with study, following the first dose of study drug; to maintain the blind, some patients receiving placebo will also have their dose modified.

**Table 1:** Glucocorticoid and matching placebo tapering schedule.

Study day	Group A: avacopan (prednisone dose, mg per day)	Group B: prednisone (control; prednisone dose, mg per day)			
	All patients	Adults		Adolescents (aged 12-17 years)	
		≥55 kg	<55 kg	>37 kg	≤37 kg
1-7	0	60	45	45	30
8-14	0	45	45	45	30
15-21	0	30	30	30	30
22-42	0	25	25	25	25
43-56	0	20	20	20	20
57-70	0	15	15	15	15
71-98	0	10	10	10	10
99-140	0	5	5	5	5
≥141	0	0	0	0	0

#### Immunosuppressive Therapy

All patients in both treatment groups also receive one of the following 3 investigator-selected baseline immunosuppressive therapy (standard-of-care) regimens:

1. Intravenous rituximab: 375 mg/m^2^ weekly × 4 infusions.
2. Intravenous cyclophosphamide: 15 mg/kg up to 1.2 g every 2 to 3 weeks for 13 weeks, and then starting on week 15, oral azathioprine 1 mg/kg daily with titration up to 2 mg/kg daily (mycophenolate mofetil 2 g daily is allowed in place of azathioprine).
3. Oral cyclophosphamide: 2 mg/kg daily for 14 weeks followed by oral azathioprine or mycophenolate mofetil starting at week 15 (same dosing regimen as intravenous cyclophosphamide).

Dose reductions or adjustments in cyclophosphamide, azathioprine, and mycophenolate are allowed to conform to standard approaches to maximize safety of these medications.

#### Additional Glucocorticoids

Use of additional glucocorticoids (eg, nonstudy supplied glucocorticoids in excess of dose and schedule outlined in Table 1) is to be avoided during the study, except for cases of adrenal insufficiency. Patients currently on glucocorticoids may enroll provided they meet the following criteria: before and during the screening visit, the use of intravenous glucocorticoids does not exceed a cumulative dose equivalent of 3 g of methylprednisolone in the 4 weeks before screening or the use of oral glucocorticoids does not exceed 10 mg once daily (QD) of oral prednisone for up to 6 weeks before screening. During the screening period (≤14 days), patients will be allowed oral glucocorticoids (tapered to ≤20 mg QD prednisone equivalent by day 1). If a patient enrolls in the study while on oral glucocorticoids, this dose must be tapered to 0 mg by the end of week 4.

Patients who experience worsening of disease during the study that involves a major item in the BVAS may be treated with intravenous glucocorticoids (typically 0.5-1 g methylprednisolone per day for 3 days) or oral glucocorticoids, tapered according to the patient's condition, or both. Worsening not involving a major item in the BVAS may be treated with a short burst (≤2 weeks) of oral glucocorticoids, at a maximum daily dose of 20 mg prednisone equivalent. The use of other medications, such as additional rituximab or cyclophosphamide, is discussed with the medical monitor before implementing. The use of plasma exchange is not permitted.

Patients experiencing a relapse or worsening of disease may continue study drug treatment and should continue in the study. In patients experiencing a relapse, the study-supplied prednisone/matching placebo will be temporarily halted during the course of glucocorticoids, and if the patient’s condition stabilizes, the study-supplied prednisone/matching placebo may be restarted according to the original study visit schedule. At the discretion of the investigator, the avacopan/matching placebo may be continued during and following the treatment for the relapse.

#### Assessments, Endpoints, and Outcomes

BVAS assessments are performed at screening and weeks 4, 10, 16, 26, 39, 52, and 60. Vasculitis damage index assessments [34] are performed at screening and weeks 26, 52, and 60. HRQoL assessments are completed on day 1 and weeks 4, 10, 16, 26, 39, 52, and 60. Glucocorticoid toxicity index assessments are performed on day 1 and at weeks 13 and 26. If a patient consents, optional renal biopsies for histology are collected at baseline if not already on file, and at week 52 or at the time of treatment discontinuation. Physical examinations, vital sign assessments, and electrocardiogram measurements are performed throughout the study. Concomitant medication and adverse events are assessed at every study visit (Multimedia Appendix 1). Determination of whether a patient enters remission and sustains remission, as well as all relapses, is assessed by an independent blinded adjudication committee.

The key clinical endpoints and outcome measures are summarized in Textboxes 3 and 4. The overall efficacy hypothesis in this study is that avacopan, in combination with immunosuppressive therapy, will be at least as effective for treatment of ANCA-associated vasculitis when compared with prednisone with immunosuppressive therapy, and may reduce the overall burden of disease by achieving remission with less exposure to glucocorticoids, fewer glucocorticoid-associated adverse effects, and improved quality of life.

**Main clinical endpoints of this study.**

- Proportion of patients in remission at week 26; defined as Birmingham Vasculitis Activity Score=0 and not taking glucocorticoids for antineutrophil cytoplasmic antibody (ANCA)–associated vasculitis within 4 weeks before week 26
- Proportion of patients achieving sustained remission at week 52; defined as remission at week 26 and week 52, without relapse through week 52, and not taking glucocorticoids for ANCA-associated vasculitis within 4 weeks before week 52

**Key outcome measures of the study.**

- Safety: adverse events, physical exam, vital signs, serum chemistry, hematology, urinalysis, and electrocardiogram
- Change in glucocorticoid-induced toxicity measured using the change from baseline in the glucocorticoid toxicity index version 2 cumulative worsening score and aggregate improvement score; cumulative use of glucocorticoids will also be analyzed. Rapidity of response based on early remission (Birmingham Vasculitis Activity Score [BVAS]=0) at week 4
- Change from baseline in health-related quality of life scores over 52 weeks based on the short form 36 version 2 and the Euro Quality of Life
- Proportion of patients and time to disease relapse after previously having achieved remission at week 26; relapse is defined as ≥1 major item in BVAS, ≥3 minor items in BVAS, or 1 or 2 minor items in BVAS at 2 consecutive visits
- Change in damage from baseline over 52 weeks, as measured by the Vasculitis Damage Index
- In patients with renal disease at baseline, change over 52 weeks in estimated glomerular filtration rate, urinary albumin: creatinine ratio, and urinary monocyte chemoattractant protein-1:creatinine ratio

### Statistical Analysis

A minimization algorithm is used to assign patients dynamically to a treatment group, which seeks to balance treatment groups with respect to each stratification factor. Statistical analysis for categorical endpoints will be expressed as the number and percentage of unique patients for each category. For continuous variables, numbers, means, medians, ranges, SDs, and standard error of means will be calculated. Geometric means will be calculated for data that are not normally distributed. Results will be presented by treatment group and by stratum for each of the 3 stratification factors. Results will be presented for patients with and without renal involvement of disease at baseline.

To estimate sample size, a noninferiority margin of −20% was based on a thorough review and meta-analysis of all previous trials conducted in patients with ANCA-associated vasculitis as well as precedent. The proportion of patients with remission in the control group at week 26 was estimated to be 60%, which is based on a blended proportion of 64% and 53% observed in the rituximab and cyclophosphamide/azathioprine groups, respectively, in the RAVE study [10]. On the basis of these assumptions and a 2-sided significance level of 0.05, a sample size of 150 patients per group provides >90% power for the noninferiority analysis. For the determination of the primary efficacy endpoints, the proportion of patients achieving disease remission at week 26 and sustained disease remission at week 52, the 2-sided 95% CIs for the difference in proportions (avacopan group minus prednisone group) will be estimated. For the noninferiority tests at week 26 and week 52, if the lower bound of the 95% CI is greater than −0.20, the avacopan group will be considered not inferior to the control group. For the superiority test, if the lower bound of the 95% CI is greater than 0.0, the avacopan group will be considered superior to the control group.

The two primary endpoints will be tested sequentially using a gatekeeping procedure to preserve the type I error rate at 0.05. The sequence of testing will be as follows:

1. Test for noninferiority of the avacopan group compared with the control group regarding remission at week 26; if the *P* value for noninferiority is *P*<.05, proceed to step 2.
2. Test for noninferiority of the avacopan group compared with the control group regarding sustained remission at week 52; if the *P* value for noninferiority is *P*<.05, proceed to step 3.
3. Test for superiority of the avacopan group compared with the control group regarding sustained remission at week 52; if the *P* value for superiority is *P*<.05, proceed to step 4.
4. Test for superiority of the avacopan group compared with the control group regarding remission at week 26.

Continuous variables of the secondary efficacy endpoints will be analyzed using a mixed effect model for repeated measures with treatment group, visit, treatment-by-visit interaction, and randomization strata as factors, and baseline as the covariate. Patients will be considered as repeated measure units over visits.

### Ethics and Dissemination

Each study site is required to obtain prior approval from an Institutional Review Board on both the protocol and patient informed consent before study initiation and in accordance with International Council for Harmonisation of Technical Requirements for Pharmaceuticals for Human Use guidelines and the Declaration of Helsinki (amended by the 59th World Medical Association General Assembly, October 2008). Safety data and efficacy outcomes data are summarized and reviewed by an unblinded Data Monitoring Committee, regularly over the course of the study.

### Patient and Public Involvement

Patients or the public were not involved in the *design* of this trial. However, patients, through a collaboration with the Vasculitis Foundation, the leading international vasculitis patient advocacy group, were involved in increasing awareness of the trial (*dissemination*) within the vasculitis patient community. It is anticipated that patients will be involved disseminating the results of this research to other patients with vasculitis, once the trial is complete and the results are made public.

## Results

The last patient was enrolled in this trial in Q4 2018. Topline results are anticipated to be published by Q3 2020.

## Discussion

### Overview

The current therapy for ANCA-associated vasculitis improves patients’ outcomes in terms of signs and symptoms of active vasculitis, but the burden of disease remains high with significant comorbidity and damage because of both glucocorticoids and disease. There is a role for targeted and less toxic treatment regimens [35]. There is increasing evidence that complement activation appears to play an important role in the pathogenesis of ANCA-associated vasculitis whereby C5aR activation augments neutrophil priming by ANCA, and directly acts as a key mediator of organ damage [4,36]. Promising results from two phase 2 studies of 12-week duration in anti-MPO- and anti-PR3-positive ANCA-associated vasculitis suggest that avacopan is well tolerated [29,30] and that its use in combination with either cyclophosphamide or rituximab may result in comparable clinical effectiveness (as assessed by the BVAS) with that of the standard-of-care glucocorticoid-containing regimen, with more rapid onset of disease control measured at week 4, as well as improved renal outcomes and quality of life [29]. Building on these observations, this larger phase 3 trial, with 1-year treatment duration and observation, aims to provide evidence for the effectiveness of C5aR inhibition for the treatment of ANCA-associated vasculitis, with potential to fundamentally change the treatment paradigm of MPA and GPA.

### Study Design Features

Given the heterogeneous clinical presentation of patients with ANCA-associated vasculitis, a large sample size is planned for this study to provide an adequate number of subjects across subgroups. Moreover, the 2 treatment groups are further balanced through stratification according to three key factors that could potentially influence patient outcomes, including ANCA subtype, new or relapsing disease, and baseline immunosuppressive therapy.

This study includes a broad range of newly diagnosed or relapsing patients with ANCA-associated vasculitis. The use, if warranted, in both treatment groups, of a limited amount of nonprotocol-specified glucocorticoids is allowed at baseline, as is the use of *rescue* glucocorticoids during the study. However, the protocol specifies the indication, dose, duration, and tapering schedule for nonprotocol-supplied glucocorticoids to limit the impact of their use on the study results.

The study incorporates frequent monitoring over the 1-year treatment period, along with a pharmacodynamic sampling program. Exploratory analyses may provide information on factors that impact rates and timing to disease remission, as well as the risk of relapse among the two treatment arms.

### Outcome Measures Selection

#### Disease Activity

This study will evaluate outcome measures at both 26 and 52 weeks along with changes over that period. The primary outcome measure of disease activity (remission) will be assessed using the BVAS version 3, the most widely accepted validated measure of disease activity and part of the Outcome Measures in Rheumatology core set of outcome measures for use in clinical trials of ANCA-associated vasculitis [37]. In this study, a team of blinded experts will adjudicate BVAS results reported by the investigators to determine if remission and sustained remission, as defined in the protocol, are attained. Slight modifications were made to the BVAS scoring criteria to suit this trial. First, all items will be scored as “new/worse” whenever they appear during the trial. Second, chronic active items will not be scored as “persistent” (persistent option removed) but are scored as per other items of activity. Third, “red blood cell casts and/or glomerulonephritis” were specified as “Other” to prompt the investigator. Fourth, for *only* the week 4 BVAS assessments, items active in the previous 7 days will be scored, not the usual 28 days.

#### Disease Impact

A major objective of this study will be to show the benefit of a treatment regimen for ANCA-associated vasculitis with greatly reduced cumulative dose of glucocorticoids. To assess the impact of reducing glucocorticoids, a glucocorticoid toxicity index will be used [33]. The Vasculitis Damage Index is used to document damage arising from disease and from treatment [37]. Activity of renal disease and preservation of renal function will be assessed by renal function measurements (glomerular filtration rate, urinary albumin:creatinine ratio, and urinary MCP-1:creatinine ratio).

Both clinical practice and trial data consistently demonstrate that HRQoL is impaired among patients with ANCA-associated vasculitis [37]. In a phase 2 study, HRQoL, including physical and mental health, was improved with avacopan use versus control [29]. The same instruments are used in this phase 3 study to ascertain any potential treatment differences in HRQoL. It is anticipated that the results from this study will contribute significantly to the understanding of HRQoL in relation to treatment for this patient population.

## Appendix

Multimedia Appendix 1 Avacopan Development in Vasculitis to Obtain Corticosteroid Elimination and Therapeutic Efficacy trial design summary.

## Abbreviations

ANCA: antineutrophil cytoplasmic antibody
BVAS: Birmingham Vasculitis Activity Score
EQ-5D-5L: Euro Quality of Life
GPA: granulomatosis with polyangiitis
HRQoL: health-related quality of life
MPA: microscopic polyangiitis
MPO: myeloperoxidase
PR3: proteinase 3
QD: once daily
SF-36: Short-Form 36
