# Supplementary material for: Evaluation of the Safety and Efficacy of Avacopan, a C5a Receptor Inhibitor, in Patients With Antineutrophil Cytoplasmic Antibody–Associated Vasculitis Treated Concomitantly With Rituximab or Cyclophosphamide/Azathioprine: Protocol for a Randomized, Double-Blind, Active-Controlled, Phase 3 Trial
Source: JMIR Res Protoc. 2020 Apr 7;9(4):e16664. doi: 10.2196/16664 (PMC7175182; doi:10.2196/16664)
Supplement: Multimedia Appendix 1 [file resprot_v9i4e16664_app1.pdf]

Multimedia Appendix 1. ADVOCATE Trial Design Summary.

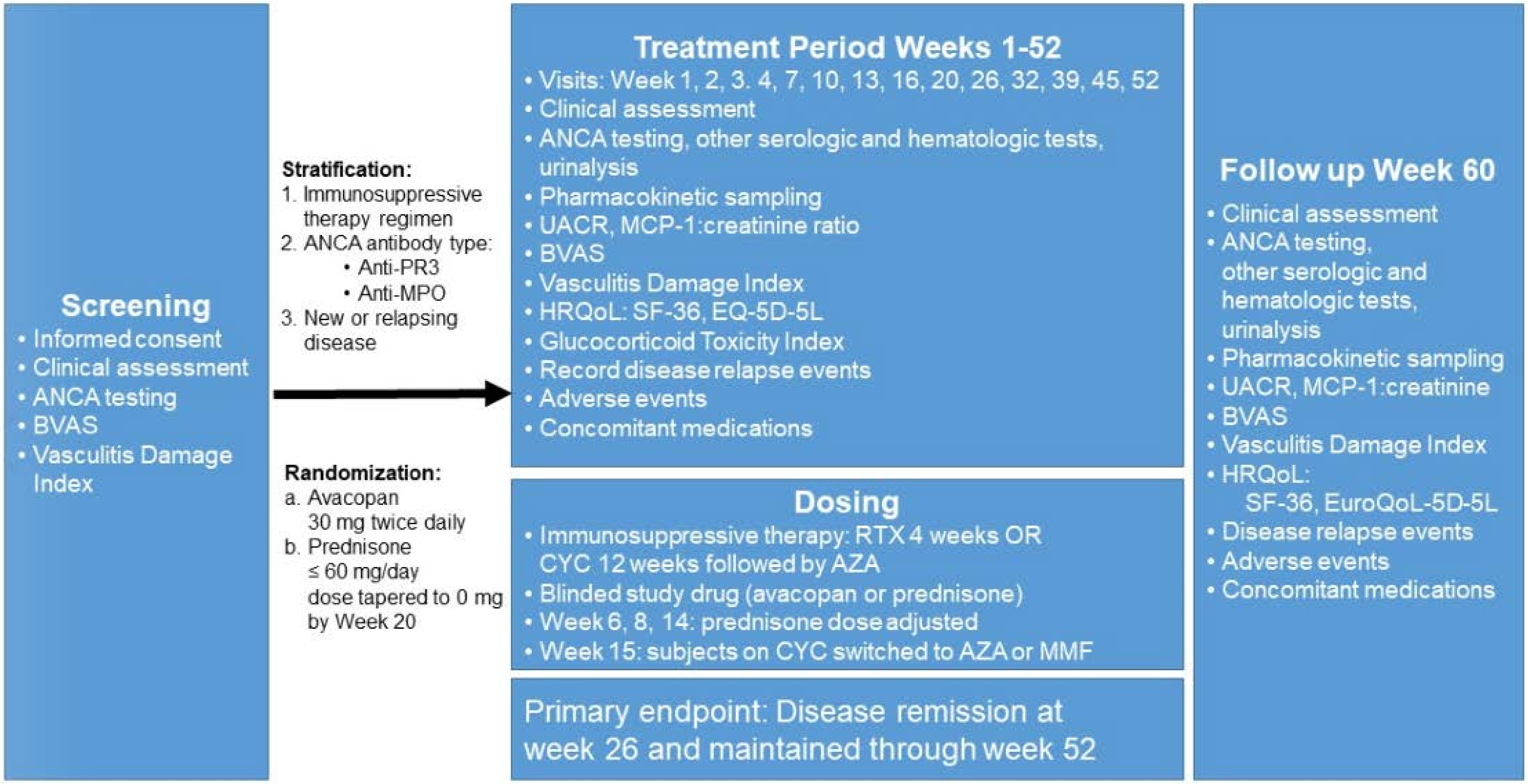

ANCA = Anti-neutrophil cytoplasmic antibody; AZA = azathioprine; BID = twice-a-day; BVAS = Birmingham Vasculitis Activity Score; CYC = cyclophosphamide; HRQoL = Health related quality of life; MCP-1 = monocyte chemoattractant protein-1; MMF = mycophenolate mofetil; MPO: myeloperoxidase; PR3: proteinase-3; RTX = rituximab; SF-36 = Medical Outcomes Study 36-item Short-Form; UACR = urinary albumin:creatinine ratio;
